# Airborne Particulate Matter and SARS-CoV-2 Partnership: Virus Hitchhiking, Stabilization and Immune Cell Targeting — A Hypothesis

**DOI:** 10.3389/fimmu.2020.579352

**Published:** 2020-09-24

**Authors:** Z. Shadi Farhangrazi, Giulio Sancini, A. Christy Hunter, Seyed Moein Moghimi

**Affiliations:** ^1^S. M. Discovery Group Inc., Denver, CO, United States; ^2^S. M. Discovery Group Ltd., Durham, United Kingdom; ^3^Department of Experimental Medicine, University of Milano-Bicocca, Monza, Italy; ^4^School of Pharmacy, College of Science, University of Lincoln, Lincoln, United Kingdom; ^5^School of Pharmacy, Newcastle University, Newcastle upon Tyne, United Kingdom; ^6^Translational and Clinical Research Institute, Faculty of Health and Medical Sciences, Newcastle University, Newcastle upon Tyne, United Kingdom

**Keywords:** alveolar macrophage, COVID-19, dendritic cell, immunity, particulate matter, pulmonary delivery, SARS-CoV-2, trained immunity

It is widely assumed that the spread of severe acute respiratory syndrome coronavirus-2 (SARS-CoV-2) infection in humans occurs through close contact with an infected person, short-range transmission through respirable droplets from an infected individuals' cough or sneeze, and aerosolized airborne droplets in long-range (over a few meters) transmission (1). Large respirable droplets (>5 μm) rapidly settle out of the air, whereas virus-laden small droplets (<5 μm), often referred to as “droplet nuclei” remain longer in the air and propagate depending on air-flow (1). Assuming that a fraction of aerosols remains infective, “droplet-nuclei” might contribute to airborne transmission of the virus, particularly in poorly ventilated and crowded indoor spaces. A recent commentary, and supported with >200 signatories, has further stressed the importance of inhalation exposure to viruses in respirable droplets at short to medium distances (up to several meters) (2).

In contrast to the inhalation mode of viral transmission through airborne respirable droplets, here we speculate an additional role for settled and airborne particulate matter (PM) not only in viral transmission through inhalation and ingestion, but also in promoting immunity through antigen delivery, adjuvanticity and trained immunity.

## PM-mediated Viral Transmission

A number of recent studies have suggested some correlations between air pollution and coronavirus disease-2019 (COVID-19) cases and deaths (3, 4). For instance, a recent epidemiological study concluded that an increase of 1 μg/m^3^ in long-term exposure to fine PM air pollutants (≤2.5 μm, PM_2.5_) is associated with an 8% increase in COVID-19 mortality rate in the United States (3). Another study involving 355 municipalities in the Netherlands has further shown that a 1 μg/m^3^ increase in concentrations is associated with 9.4 more COVID-19 cases, 3.0 more hospital admissions, and 2.3 more deaths (4). Similarly, previous reports have also indicated that air pollution exposure increases severe outcomes during infectious disease outbreaks (5, 6). For instance, during an outbreak in 2003 in China, severe acute respiratory syndrome case fatality rates were dramatically higher in locations with a moderate to high long-term air pollution index in comparison to regions with a low air pollution index (5). While long-term exposure to air pollutants such as PM_2.5_ and nitrous dioxide contributes to persistent inflammatory responses and cardiopulmonary diseases (7), which might increase vulnerability to COVID-19, it is also plausible that depending on the environment SARS-CoV-2 “hitchhiking” on airborne PM pollutants might be an additional mechanism for spreading the infection. A number of studies have shown that PM is a carrier of airborne pathogens (8, 9). For instance, a metagenomic study has confirmed the presence of bacteria, archaea, fungi and dsDNA viruses on PM pollutants collected during a severe smog event in Beijing (8). More recently, Setti et al. (10) detected the presence of SARS-CoV-2 RNA on outdoor/airborne PM of 2.5–10 μm (PM_10_) collected from an industrial site of Bergamo Province, which is known for high air pollution and some of the most severe cases of COVID-19. This study was limited to detection of viral nucleic acid, rather than infectious virus. However, it is likely that air samplers can severely damage pathogens making detection of intact microorganisms difficult. Notwithstanding, the exact role of PM in transmission of pathogens remains to be elucidated, but will be dependent on the presence of bound/trapped infectious viruses.

**Figure 1:**
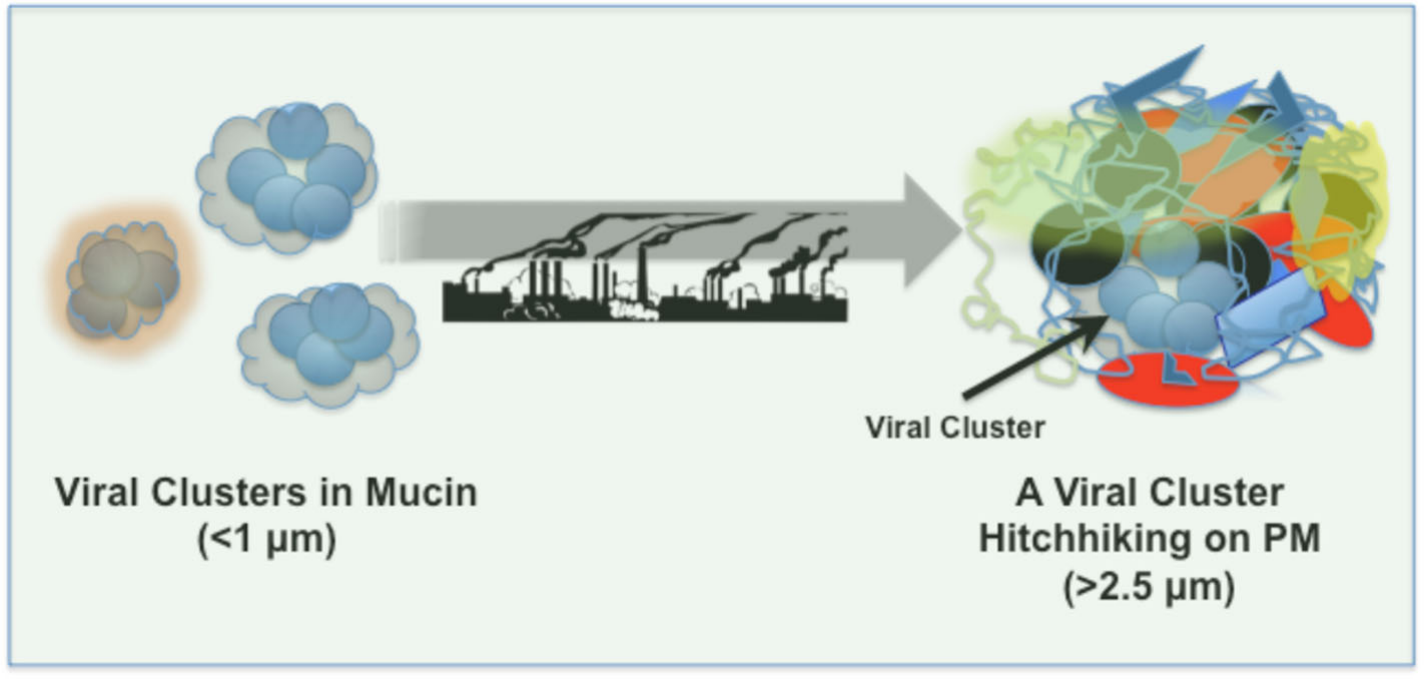
SARS-CoV-2 clusters in mucin and deposition on airborne PM pollutants.

Can infectious viruses survive on PM? In “droplet nuclei” SARS-CoV-2 remain stable for at least up to 3 h, whereas on plastic and stainless steel surfaces the viral stability is increased dramatically and detectable up to 72 h after application (11). Viruses in aerosol droplets, originated from a cough or sneeze, are likely embedded in and covered by a “corona” of mucins/proteoglycans (Figure 1), which may aid viral aggregation and preservation as well as viral adsorption to airborne PM and/or settled PM on surfaces. Furthermore, PM, depending on its composition, shape, physicochemical properties and density might further aid viral clustering and preservation, and account for prolonged viral stability and persistence on surfaces and in the air. Viral adhesion might also bridge PM-PM binding/agglomeration and enhance respirable properties of these structures.

**Figure 2:**
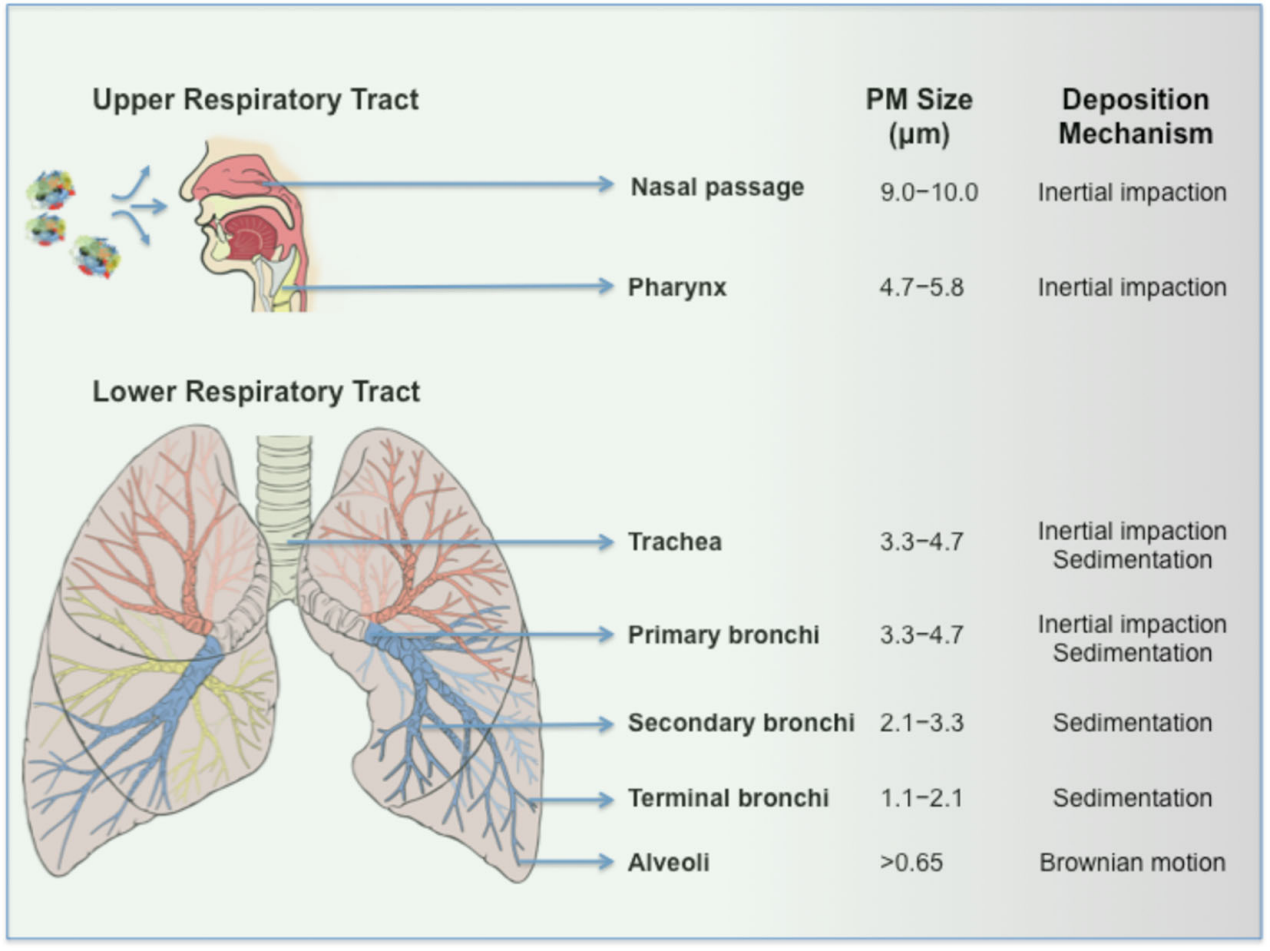
Inhalation of SARS-CoV-2-PM and their respiratory tract deposition.

Can PM-virus composites induce infection? Viruses bound to PM seemingly resemble drug powders adsorbed to coarse carrier particles (e.g., lactose) in dry powder inhalers (DPI) in their behavior. In DPI, coarse carriers increase drug particle dispersion, improve flow and on inertial impaction in the back of the throat releases drug particles, which subsequently deposit to the lower respiratory tract regions by sedimentation (12). On inhalation, infectious virus clusters bound to PM (e.g., composites >5 μm) are expected to predominantly deposit in the upper respiratory airways due to greater inertia (Figure 2). The binding target for SARS-CoV-2 is angiotensin-converting enzyme 2 (ACE2), which is not only expressed by lung epithelial cells in the lower respiratory tract (13), but also in the nose as reported recently (14, 15), as well as proximal and distal enterocytes (16). Assuming that some bound viruses to PM remain intact and infectious, the bulk of inhaled virus-bound PM most likely corresponds to a very low viral titer. In spite of this, PM could increase viral persistence in the upper respiratory airways and through irritation and ulceration of the nasal epithelium (14, 15) promotes viral spread particularly in individuals where mucociliary clearance is reduced (e.g., smokers, asthma, acute respiratory distress syndrome). It has been recently reported that inhalation of as few as 50 viral RNA copies are enough to effectively induce disease and this supports the notion that the inefficient nature of particulate inhalation could still achieve pathogenesis (17). Accordingly, this mode of inhalation might eventually promote viral translocation to the lower respiratory tract and contribute to disease severity (Figure 2). Second, those inhaled large-sized virus-bound PM in the mouth and throat are susceptible to ingestion. It is also plausible that PM adsorption/embedding might enhance viral stability in the hostile environment of the gastrointestinal tract and eventually promotes infection through interaction with ACE2 expressing enterocytes (or even through sampling by M cells in the gut-associated lymphoid tissue). Third, considering the complex and composite nature of airborne PM (which is typically an agglomerate of carbonaceous combustion particles, coarse dust, fibers, microplastics, transition elements, secondary nitrates, sulfates, adsorbed gases, etc.) (7, 18), inertial compaction may de-agglomerate PM forming smaller viral-bound particles. Virus-PM composites of <5 μm, depending on their shapes and densities, could distribute to primary, secondary and terminal bronchi as well as reach the alveoli by sedimentation. Indeed, these are anatomical regions where significant inflammatory reactions have been observed in COVID-19 cases (19). Within the alveoli, instead of targeting ACE2 expressing epithelial cells, virus-PM composites could be highly susceptible to phagocytic recognition and clearance by alveolar macrophages (AM) through a plethora of endocytic and pattern-recognition receptors as well as ACE2 (which is also expressed by AM), where even a low dose exposure might result in macrophage infection. However, this still requires pathogen translocation from endolysosomal compartments to the cytosol and successful viral replication. Earlier studies have established a cytoplasmic mode of entry for a predecessor coronavirus through a proteolysis-dependent endo-lysosomal pathway (20), which might apply to SARS-CoV-2. Although coronaviruses such as SARS-CoV replicate poorly in human monocytes/macrophages (21), shedding of low viral titres from a small population of AM might still be sufficient to spread infection through the regional ACE2 expressing epithelial cells. Furthermore, by considering the heterogeneous composition and proinflammatory nature of airborne PM pollutants, on phagocytosis, some PM might act synergistically with viruses to initiate the bystander alveolar macrophage-mediated damage to epithelial tissues. For instance, this could occur through a cytokine storm and coupled with down-regulation of CD200R (a receptor which inhibits macrophage activation) and up-regulation of tumor necrosis factor-related apoptosis-inducing ligand (TRAIL) on the AM surface (22). Subsequently, TRAIL binding to death receptor 5 (DR5) expressed on epithelial cells could induce apoptosis in the epithelium (22) resulting in alveolar leakage and spread of virus out of the affected alveoli. Furthermore, these disruptions through PM-virus alliance may further help with the recruitment and activation of monocyte-derived macrophages that is seen in patients with COVID-19 (23).

In summary, although long-term exposure to polluted air might increase vulnerability to COVID-19 through prior adverse cellular effects of settled PM (24), our proposed “hitchhiking” hypothesis offers an additional multi-mechanistic pathogenic process through delivery of low viral titres with diverse PM-virus composites and is applicable to both indoor and outdoor situations, where the pathogenic severity is dependent on PM concentration, composition, shape and size as well as the infectious viral load. For instance, the hypothetical concentration of viruses carried by PM_2.5_ is expected to be much lower than those by PM_10_, since the carrying capacity is proportional to the particle volume. Accordingly, larger PM might play more significant roles in viral transmission. Nevertheless, it is still plausible that during infectious disease outbreaks pathogen hitchhiking on PM_2.5_ might be an additional, and yet, effective contributing factor to epidemic and disease pathogenesis in urban as well as rural hot spots (e.g., locations with intensive livestock farming, which is rich in PM_2.5_) with poor local air pollution and particularly among high-risk individuals.

## PM Contribution to Immunity

Contrary to the suggestions that long-term exposure to PM might increase vulnerability to SAR-CoV-2 infection, inhaled PM might promote some forms of immunity to the virus in some individuals. PM could be the carrier of spike, envelope, membrane and nucleocapsid protein fragments of coronaviruses and on inhalation could deliver accompanied antigens to a variety of dendritic cells (DCs) subsets located throughout the respiratory tract. Since, a large number of DCs are associated with the large airways (e.g., in the respiratory epithelium of the nose, nasopharynx, trachea, and large bronchi) (25), these DCs might recognize and capture inhaled PM-antigen composites through receptor-mediated endocytic processes. Depending on PM composition, physicochemical characteristics and antigen load they could undergo a maturation process, leave the lung, and migrate to draining lymphoid tissues, where they are capable of activating naïve T cells for the expression of acquired immunity. Thus, PM could not only act as an antigen depot, but also as an adjuvant to initiate DC maturation. On the other hand, it is even more tempting to speculate that frequent exposure to particular types of PM/PM-antigen composites (e.g., as in areas with high air pollution) could trigger some forms of trained immunity (a de facto innate immune memory) presumably through epigenetic reprogramming of transcriptional pathways (26), thus offering a plausible explanation as to why some SARS-CoV-2 infected individuals are asymptomatic. Interestingly, evidence suggests that the asymptomatic COVID-19 patients have a significantly lower virus-specific IgG and neutralizing antibody levels relative to symptomatic patients in the early convalescent phase (27). Furthermore, asymptomatic individuals exhibited lower levels of many pro- and anti-inflammatory cytokines (27). These observations may be indicative of trained immunity in these individuals. Irrespective of the immunological outcomes, these possibilities are analogous to immunization strategies with engineered nano- and micro-particles (28).

## Conclusions

Here, we propose a hypothesis and a working mechanism for the role of PM pollutants in binding, stabilization and delivery of low titres of SARS-CoV-2 to alveolar macrophages. Perturbations of macrophage function by virus-PM composites may be an additional mechanism for spreading infection and contributing to COVID-19 severity. Contrary to this, some inhaled PM might promote immunity to SARS-CoV-2 through complex mechanisms. Since, the role of PM in airborne transmission of pathogens and associated immune responses are poorly understood, research in this neglected area should be encouraged, resulting in understanding that might contribute to better prevention of respiratory diseases and spread of drug-resistant respiratory pathogens in the future.

## Author Contributions

All authors listed have made a substantial, direct and intellectual contribution to the work, and approved it for publication.

## Conflict of Interest

ZF is the CEO of S. M. Discovery Group Inc. and S. M. Discovery Group Ltd. The remaining authors declare that the research was conducted in the absence of any commercial or financial relationships that could be construed as a potential conflict of interest.
